# Prevalence of prehypertension and its risk factors in midlife and late life: Indonesian family life survey 2014–2015

**DOI:** 10.1186/s12889-021-10544-y

**Published:** 2021-03-12

**Authors:** Aida Lydia, Siti Setiati, Czeresna Heriawan Soejono, Rahmi Istanti, Jessica Marsigit, Muhammad Khifzhon Azwar

**Affiliations:** 1 Department of Internal Medicine, Division of Nephrology and Hypertension, Cipto Mangunkusumo Hospital, Faculty of Medicine Universitas Indonesia, Jakarta, Indonesia; 2 Clinical Epidemiology and Evidence-Based Medicine, Cipto Mangunkusumo Hospital, Jakarta, Indonesia; 3 Department of Internal Medicine, Division of Geriatric Medicine, Cipto Mangunkusumo Hospital, Faculty of Medicine Universitas Indonesia, Jakarta, Indonesia; 4 Faculty of Medicine Universitas Indonesia, Jakarta, Indonesia

**Keywords:** Prehypertension, Risk factors, Indonesia, Mid- and late-life

## Abstract

**Background:**

Early detection of prehypertension is important to prevent hypertension-related complications, such as cardiovascular disease, cerebrovascular disease and all-cause mortality. Data regarding the prevalence of prehypertension among mid- and late-life population in Indonesia were lacking. It is crucial to obtain the prevalence data and identify the risk factors for prehypertension in Indonesia, which may differ from that of other countries.

**Methods:**

The cross-sectional analysis utilized multicenter data from Indonesian Family Life Survey-5 (IFLS-5) from 13 provinces in 2014–2015. We included all subjects at mid-and late-life (aged ≥40 years old) from IFLS-5 with complete blood pressure data and excluded those with prior diagnosis of hypertension. Prehypertension was defined as high-normal blood pressure according to International Society of Hypertension (ISH) 2020 guideline (systolic 130–139 mmHg and/or diastolic 85–89 mmHg). Sociodemographic factors, chronic medical conditions, physical activity, waist circumference and nutritional status were taken into account. Statistical analyses included bivariate and multivariate analyses.

**Results:**

There were 5874 subjects included. The prevalence of prehypertension among Indonesian adults aged > 40 years old was 32.5%. Age ≥ 60 years (adjusted OR 1.68, 95% CI 1.41–2.01, *p* <  0.001), male sex (adjusted OR 1.65, 95% CI 1.45–1.88, *p* <  0.001), overweight (adjusted OR 1.44, 95% CI 1.22–1.70, *p* <  0.001), obesity (adjusted OR 1.77, 95% CI 1.48–2.12, *p* <  0.001), and raised waist circumference (adjusted OR 1.32, 95% CI 1.11–1.56, *p* = 0.002) were the significant risk factors associated with prehypertension. Prehypertension was inversely associated with being underweight (adjusted OR 0.74, 95% CI 0.59–0.93, *p* = 0.009).

**Conclusions:**

The prevalence of prehypertension in Indonesian mid- and late-life populations is 32.5%. Age ≥ 60 years, male sex, overweight, obesity, and raised waist circumference are risk factors for prehypertension.

## Background

In 2003, the seventh report of the Joint National Committee (JNC-7) introduced “prehypertension” as a new blood pressure (BP) classification [1]. Since then, numerous studies focused on this topic. Currently, physicians make the diagnosis of high-normal BP or prehypertension based on the findings of systolic blood pressure (SBP) being 130—139 mmHg and/or diastolic blood pressure (DBP) being 85—89 mmHg following repeated meassurement [2].

Several meta-analyses discovered that individuals with prehypertension were at higher risk of developing cerebrovascular disease (CVD), coronary artery disease (CAD), chronic kidney disease (CKD), and of fatal outcome [3–5]. In large population studies conducted in the United States and China, more than 30% of adults suffered from prehypertension [6–8]. In Saudi Arabia, more than 50% of adults had prehypertension [9]. In comparison, 37.1 and 12% of adults had prehypertension in Malaysia and Thailand, respectively [10, 11].

Little is known about the prevalence of prehypertension among mid- and late-life populations in Indonesia, although prehypertension is known to be related to CVD events and all-cause mortality. It is essential for the Indonesian government and physicians to identify the risk factors for prehypertension in the Indonesian population, which may differ from those of other countries.

To our best knowledge, to date, this is the first multicenter study in Indonesian middle-aged and older adults with large sample size to estimate the real prevalence of prehypertension at the national level.

## Methods

### Study design and subjects

This cross-sectional analysis utilized multicenter data from Indonesian Family Life Survey-5 (IFLS-5) conducted in 2014–2015 from 13 provinces, comprising four provinces on Sumatra island, five provinces on Java, and four provinces in East Indonesia. The sampling strategy stratified on provinces, then randomly within the provinces [12]. In IFLS-5, 16,204 households and 29,965 individuals aged 18 years and older were interviewed and had complete blood pressure measurements. The inclusion criteria for the cross-sectional analysis were all subjects at mid and late life (aged ≥40 years old) with complete BP data from the IFLS-5 survey [12]. We excluded all subjects who had been diagnosed with hypertension, as well as all subjects with incomplete data related to BP and other study variables.

### Variable classifications

Demographic data included age, sex, education, marital status, history of smoking, and chronic medical conditions on history taking. Participants answered several binary questions, including “Have you ever chewed tobacco, smoked a pipe, smoked self-rolled cigarettes, or smoked cigarettes/cigars?” Self-reported chronic medical condition was assessed by using the following yes-no question, “Has a doctor/paramedic/nurse/midwife ever told you that you had …? (diabetes mellitus [DM], coronary artery disease, stroke, dyslipidemia, psychiatric problem, and/or memory-related diseases).”

Fatigue and sleeping status were obtained by using rating scale questions. Individuals were asked whether they felt tired in the previous 7 days. Response was recorded as 1 = not at all, 2 = a little bit, 3 = somewhat, 4 = quite a bit, or 5 = very much. Response related to trouble sleeping in the previous 7 days was also recorded using the same scale [12].

Functional status was measured by using the Katz Index of Independence in Activities of Daily Living. It consisted of six components: bathing, dressing, toileting, getting in/out of bed, continence, and feeding. One point was given for each component if the individual managed to do the activity independently, while zero point, conversely, was given to each component if the individuals required partial or total help. The maximum score was 6, indicating that the subject was independent, whereas a score of 0 signified the dependence of the subject [12].

Physical activity was assessed with an abbreviated version of the “International Physical Activity Questionnaire (IPAQ)” short version, for the last 7 days (IPAQ-S7S). Physical activity was categorized according to the IPAQ scoring protocol as low and moderate—high [13]. Weight was measured by using a Camry model EB1003 scale with measurement to the nearest kilogram, whereas height was measured by using a Seca plastic height board model 213, measured to the nearest millimeter. Waist circumference was measured with a tape measurement to the nearest millimeter [12].

Cut-offs of > 80 cm and > 90 cm, respectively, were applied for raised waist circumference in women and men [14]. Body mass index (BMI) was classified based on Western Pacific Region of World Health Organization (WHO) criteria pertaining to obesity criteria (WPRO criteria) [15]. The categories are underweight (< 18.5 kg/m^2^), normoweight (18.5—22.9 kg/m^2^), overweight (23.0—24.9 kg/m^2^), and obesity (≥ 25.0 kg/m^2^).

Three successive measurements of systolic and diastolic BP of alternate arms were recorded with an Omron meter HEM-7203, by regular trained interviewers, and the average BP of each individual was used. The BP of eac participants was measured while seated on a chair with a prior five-minute rest. Prehypertension or high-normal BP status was based on the presence of either SBP of 130—139 mmHg and/or DBP of 85—89 mmHg according to International Society of Hypertension (ISH) 2020 guidelines [2] and Indonesian Consensus for Management of Hypertension in 2019.

### Statistical analysis

The dependent variable in this study was prehypertension status. Independent variables were sex, age, marital status, smoking status, physical activity, nutritional status, waist circumference, self-reported chronic medical condition (diabetes mellitus, coronary artery disease, stroke, dyslipidemia, psychiatric problem, memory-related disease), fatigue condition, sleep disorder, and functional status.

BP was categorized into (1) normal BP (SBP < 130 mmHg and/or DBP < 85 mmHg) or (2) Prehypertension (SBP 130—139 mmHg and/or DBP 85—89 mmHg). Age was categorized into (1) 40–59 years or (2) > 60 years. Categories according to marital status were (1) married or (2) single/widowed/divorce. Physical activity was categorized into two categories (1) moderate and high or (2) low. Nutritional status according to BMI was categorized into (1) normal, (2) underweight, (3) overweight, or (4) obesity. Waist circumference was divided into (1) normal and (2) raised. Chronic medical conditions, psychiatric problems, memory-related diseases, fatigue, and sleeping disorders were recorded as (1) yes or (2) no. Functional status was categorized into (1) independent (score 4—6) or (2) dependent (score 0—3).

We performed statistical analysis using SPSS Version 16 (IBM, Armonk, New York, USA). Descriptive analysis was done to explore the characteristics of subjects. Categorical variables were presented as number and percentage. Bivariate and multivariate logistics regression were performed to obtain crude and adjusted odds ratio of nearly all study variables. We obtained the adjusted odds ratios following adjustment of possible confounders based on the bivariate analysis result (*p*-value < 0.25).

## Results

We included complete data from 5874 subjects. We found that 32.5% of subjects had prehypertension. Among men, 35.1% of them were in a prehypertensive state, whereas only 29.4% of women had prehypertension. The mean (SD) age of all subjects was 48.8 (7.2) years, whereas the mean ages (SD) of subjects with normal BP and and of those prehypertension were 48.4 (7.1) years and 49.7 (7.5) years, respectively. Other characteristics of subjects are shown in Table 1.

**Table 1:** Characteristics of subjects

Characteristics	All subjects (***n*** = 5874)	Normal blood pressure (***n*** = 3965)	Prehypertension (***n*** = 1909)
Age			
40–59 years	5246 (89.3)	3592 (90.6)	1654 (86.6)
≥ 60 years	628 (10.7)	373 (9.4)	255 (13.4)
Sex			
Female	2708 (46.1)	1911 (48.2)	797 (41.7)
Male	3166 (53.9)	2054 (51.8)	1112 (58.3)
Marital status			
Single	113 (1.9)	76 (1.3)	37 (1.9)
Married	5153 (87.7)	3465 (87.4)	1688 (88.4)
Separated	41 (0.7)	32 (0.8)	9 (0.5)
Divorced	169 (2.9)	121 (3.1)	48 (2.5)
Widowed	398 (6.8)	271 (6.8)	127 (6.7)
Educational attainment			
Senior high school or higher education	2178 (37.1)	1494 (37.7)	684 (35.8)
Junior high school	991 (16.9)	686 (17.3)	305 (216.0)
Elementary school or no formal education	2705 (46.1)	1785 (45.0)	920 (48.2)
Body mass index			
Normal	2283 (38.9)	1659 (41.8)	624 (32.7)
Underweight	523 (8.9)	406 (10.2)	117 (6.1)
Overweight	1037 (17.6)	675 (17.0)	362 (19.0)
Obesity	2031 (34.6)	1225 (30.9)	806 (42.2)
Waist circumference			
Normal	3377 (57.4)	2406 (60.7)	971 (50.9)
High	2497 (42.6)	1559 (39.3)	938 (49.1)
Smoking history			
No	3250 (55.3)	2236 (56.4)	1014 (53.1)
Yes	2624 (44.7)	1729 (43.6)	895 (46.9)
Physical activity			
Moderate and high	2451 (41.7)	1656 (41.8)	795 (41.6)
Low	3423 (58.3)	2309 (58.2)	1114 (58.4)
Dyslipidaemia			
No	5553 (94.5)	3757 (94.8)	1796 (94.1)
Yes	321 (5.5)	208 (5.2)	113 (5.9)
Psychiatric problem			
No	5866 (99.9)	3960 (99.9)	1906 (99.8)
Yes	8 (0.01)	5 (0.1)	3 (0.2)
Sleeping disorder			
No	3437 (58.5)	2321 (58.5)	1116 (58.5)
Yes	2437 (41.5)	1644 (41.5)	793 (41.5)
Memory-related disease			
No	5868 (99.9)	3960 (99.9)	1908 (99.9)
Yes	6 (0.01)	5 (0.1)	1 (0.1)
Fatigue			
No	2261 (38.5)	1475 (37.2)	786 (41.2)
Yes	3613 (61.5)	2490 (62.8)	1123 (58.8)
Functional status			
Independent	3227 (54.9)	2220 (56.0)	1007 (52.8)
Dependent	2647 (45.1)	1745 (44.0)	902 (47.2)
Diabetes mellitus			
No	5712 (97.2)	3861 (97.4)	1851 (97.0)
Yes	162 (2.8)	104 (2.6)	58 (3.0)
Stroke			
No	5864 (99.8)	3959 (99.8)	1905 (99.8)
Yes	10 (0.02)	6 (0.2)	4 (0.2)
Coronary artery disease			
No	5750 (97.9)	3887 (98.0)	1863 (97.6)
Yes	124 (2.1)	78 (2.0)	46 (2.4)

Data were shown as n(%)

Similarly, the majority of subjects with prehypertension were 40—59 years old (86.6%), male (58.3%), married (88.4%), and only graduated from elementary school or had no formal education at all (48.2%). However, a high proportion of subjects with prehypertension were obese (42.2%), and had a normal waist circumference (50.9%), low physical activity (58.4%), fatigue (58.5%), independent functional status (52.8%), no smoking history (53.1%), no self-reported dyslipidemia (94.1%), no psychiatric problem (99.8%), no sleeping disorder (58.5%), no memory-related disease (99.9%), no self-reported DM (97.2%), no stroke (99.8%), and no coronary artery disease (97.6%).

The crude OR each factor based on bivariate analysis is shown in Table 2. Variables with *p*-value < 0.25 in bivariate analysis were included in multivariate analysis. Therefore, the included variables in multivariate analysis were age, sex, body mass index, waist circumference, and functional status; see Table 3. Following the adjustment of possible confounding factors, age ≥ 60 years (adjusted OR 1.68, 95% CI 1.41–2.01, *p* <  0.001), male sex (adjusted OR 1.65, 95% CI 1.45–1.88, *p* <  0.001), overweight (adjusted OR 1.44, 95% CI 1.22–1.70, *p* <  0.001), obesity (adjusted OR 1.77, 95% CI 1.48–2.12, *p* <  0.001), and raised waist circumference (adjusted OR 1.32, 95% CI 1.11–1.56, *p* = 0.002) were the significant risk factors associated with prehypertension in middle-aged and older people in Indonesia. Dependent functional status was not significantly associated with prehypertension in this case (adjusted OR 1.11, 95% CI 0.99–1.24, *p* = 0.085). On the other hand, prehypertension was inversely associated with being underweight (adjusted OR 0.74, 95% CI 0.59–0.93, *p* = 0.009).

**Table 2:** Crude OR of factors associated with prehypertension based on bivariate analysis

Variable	Crude odds ratio	
	OR (95% CI)	***p-***value
Age		
40–59 years	1	
> 60 years	1.49 (1.25–1.76)	0.000
Sex		
Female	1	
Male	1.29 (1.16–1.45)	0.000
Marital status		
Married	1	
Single / divorced / widowed	0.91 (0.77–1.07)	0.258
Body mass index		
Normal	1	
Underweight	0.77 (0.61–0.96)	0.021
Overweight	1.43 (1.22–1.67)	< 0.001
Obesity	1.75 (1.54–1.99)	< 0.001
Waist circumference		
Normal	1	
Raised	1.49 (1.34–1.66)	< 0.001
Smoking history		
No	1	
Yes	1.14 (1.02–1.27)	0.018
Physical activity		
Moderate and high	1	
Low	1.01 (0.90–1.12)	0.930
Dyslipidaemia		
No	1	
Yes	1.14 (0.89–1.44)	0.289
Psychiatric problem		
No	1	
Yes	1.25 (0.29–5.22)	0.762
Sleeping disorder		
No	1	
Yes	1.00 (0.89–1.21)	0.955
Memory-related disease		
No	1	
Yes	0.42 (0.15–3.56)	0.407
Fatigue		
No	1	
Yes	0.85 (0.76–0.95)	0.003
Functional status		
Independent	1	
Dependent	1.14 (1.02–1.27)	0.019
Diabetes mellitus		
No	1	
Yes	0.16 (0.84–1.61)	0.363

**Table 3:** Adjusted OR of factors associated with prehypertension based on multivariate analysis

Variable	Adjusted odds ratio	
	OR (95% CI)	***p***-value
Age		
40–59 years	1	
> 60 years	1.68 (1.41–2.01)	< 0.001
Sex		
Female	1	
Male	1.65 (1.45–1.88)	< 0.001
Body mass index		
Normal	1	
Underweight	0.74 (0.59–0.93)	0.009
Overweight	1.44 (1.22–1.70)	< 0.001
Obesity	1.77 (1.48–2.12)	< 0.001
Waist circumference		
Normal	1	
Raised	1.32 (1.11–1.56)	0.002
Functional status		
Independent	1	
Dependent	1.11 (0.99–1.24)	0.085

Mutually adjusted odds ratios (with 95% confidence intervals) from the result of multivariate analysis including all variables for which results are presented in this table

## Discussion

### Prevalence of prehypertension among Indonesian middle-aged and older adults

The prevalence of prehypertension in this study was 32.5%. Similarly, the prevalence of prehypertension among middle-aged and older Taiwanese population was 35.8% [16]. A study in young adults conducted in Indonesia also showed a similar prevalence of prehypertension (34.2%) [17]. We found only 29.4% prevalence of prehypertension among middle-aged and older females in Indonesia. In contrast, a study conducted in China among 16,981 aging women concluded that 47% of subjects had prehypertension [18]. The findings suggest that prehypertension is common among mid- and late-life populations in Indonesia, as well as in other Asian countries.

### Older age and prehypertension

According to the results of multivariate analysis, older age was an important associated factor in prehypertension. People aged 60 years and older were found to have 1.7 times higher risk of having prehypertension compared with those aged 40—49 years (*p* <  0.001). This finding is in conformance with the result of a cross-sectional study in Malaysia showing the odds of developing prehypertension to be 1.06 times higher with each 1-year increment of age (adjusted OR 1.06, 95% CI 1.02—1.11, *p* = 0.007) [10]. The association between age and trends in BP has long been known [19, 20]. In general, there is a positive correlation between age and both SBP and DBP [14]. Arterial stiffness increases during the aging process and increases synchronously with increasing BP. Therefore, recommending the adoption of strategies that promote healthy vascular aging strategies may be helpful in delaying age-associated increases in arterial stiffness and BP. These include physical activity (e.g., aerobic vs resistance exercise), weight loss or decreased total energy intake, composition of diet (e.g., diet rich in flavonoids), and pharmacological agents [21, 22].

### Men and prehypertension

Our study showed that 58.3% of prehypertensive individuals were men, and men had a 1.7 times higher risk of developing prehypertension compared with women. Previous studies in Saudi Arabia, India, and Southern China also suggested similar conclusions [9, 23, 24]. Typically men had higher BP and developed cardiovascular disease earlier than women [25]. Although not fully understood, this is possibly due to differences in hormonal activity in both groups [26].

The mean age (SD) of subjects in this study was 48.8 (7.2) years. Hence, some of the women in this study might not have reached menopause and thus the women tend to have lower level of BP due to the protective effect of estrogen [27]. After menopause, women may outnumber men in the prevalence of hypertension. Loss of estrogen at any age may also contribute to the endothelial dysfunction commonly found in hypertensive patients [28].

### High BMI, raised waist circumference, and prehypertension

Both overweight and obesity in Indonesian adults aged ≥40 years were associated with prehypertension in this study. Despite the BMI cut-off difference used compared with our study, a study in Saudi Arabia by Aldiab and colleagues also found an association between abnormally higher BMI (overweight and obesity) and prehypertension [9]. This study result also supports the data from other developed and developing countries related to the strong relationship between BP and BMI [29]. Obesity has been positively associated with insulin resistance. Insulin contributes to the regulation of BP by stimulating the production of nitric oxide (NO) in endothelium inducing vasodilation and enhancing sodium reabsorption in the kidney [30]. Thereby, insulin resistance is involved in the development of cardiovascular diseases and hypertension.

Each year there is an increasing trend toward obesity in Indonesia [31]. This finding suggests that the prevalence of prehypertension will continue to grow if there is no specific management of excess body weight. Body weight control is indicated to avoid obesity in adults. A review in South Africa suggested that combined exercise and weight-loss intervention has been shown to decrease SBP by more than 10 mmHg. There is also evidence to suggest that exercise training and weight loss are associated with improvement in endothelial function, left ventricular structure and function, as well as reduction in arterial stiffness [32].

Waist circumference is related to the percentage of abdominal fat mass [32]. An association between raised waist circumference and prehypertension was found in this study. Abnormal waist circumference is also associated with prehypertension among Malaysian adults (adjusted OR 31.65, 95% CI 11.25—89.02, *p* <  0.001) [10]. Indonesia’s quinquennial national health registry data also revealed an increasing trend of raised waist circumference among citizens aged 15 years and older. There were 18.8 and 26.6% of citizens aged 15 years or older with central obesity/raised waist circumference in the year 2007 and 2013, respectively. Interestingly, about one in three Indonesians had documented raised waist circumference in 2018 (based on Indonesian National Health Research) [31].

Evidence supported that the accumulation of fat in the abdominal area (central obesity) is a greater risk factor for cardiovascular disease than other types of obesity [33]. Therefore, it is important for physicians to know the distribution of fat, more than simple nutritional status based on BMI, in order to understand each individual’s risk of cardiovascular diseases [33]. For example, people with normal BMI may have raised waist circumference. Both risk factors can be related to prehypertension independently [10].

### Underweight and prehypertension

Being underweight appears to be protective against prehypertension in our study (adjusted OR 0.74, 95% CI 0.59–0.93, *p* = 0.009). Previously, evidence suggested a steady increase in blood pressure from being underweight to being of normal weight and further to being overweight/obese among Bengali adults [34]. It is hypothesized that being underweight in middle-aged and older adults is protective against prehypertension through lower fat and carbohydrate intake. Approach to weight loss in older adults, however, should include a risk—benefit review of the strategy related to the danger of sarcopenia, higher risk of hip fracture, and increased mortality [35].

### Clinical implications of the results

Prehypertension may act as an alarm to all physicians to start the prevention of its progression to hypertension and its cardiovascular complications [36]. In addition, such normal-high BP is a known risk factor for masked hypertension [37]. Analysis of data from the Framingham Heart Study cohort showed that the rate of progression from prehypertension becoming hypertension was estimated to be 19% in 4 years [36]. Of major importance, the presence of atherosclerotic cardiovascular disease (ASCVD) and an average SBP of ≥130 mmHg or an average DBP of ≥80 mmHg; or a patient’s result of calculated 10-year ASCVD risk of ≥10% and an average SBP of ≥130 mmHg or an average DBP of ≥80 mmHg should guide the physician to start BP-lowering drug treatment as recommended by the American College of Cardiology/American Heart Association in 2017 [38].

The increasing trend in elevated BP between 1990 and 2015 in the world as shown by data from 154 countries was also accompanied by a substantial change in overweight and obesity, fruit and vegetable consumption, physical activity, and dietary salt intake over the same period of time. Hence, the prevention and control of high BP through a combination of pharmacological and/or nonpharmacological strategies could mitigate the growing burden associated with high SBP [39]. In individuals with prehypertension, lifestyle modification is also the cornerstone of management. Healthy lifestyle choices can prevent and delay the onset of high BP. Healthy diet, moderation of alcohol consumption, weight reduction, smoking cessation, regular physical activity, stress reduction, and salt reduction are shown to be key factors in an effective health policy regimen [2].

Among prehypertensive individuals recruited into the randomized clinical trial, mindfulness-based stress reduction as antistress therapy resulted in significantly lower office BP compared with progressive muscle relaxation training [39]. Older people may not reach the target of physical activity recommended for young adults. Thus, physical activity in older people should consider the functional capacity and the preference of each person [40].

A low-sodium diet is inversely associated with clerical work jobs, frequent eating out, and 40—49 years age group. Even after adjustment for potential confounding factors, lower frequency of eating out (≤ 3 times/month) is significantly associated with low-sodium diet compared with individuals with the habit of eating out on a daily basis (< 1 time/month: OR 1.97, 95% CI 1.49—2.61; 1—3 times/month: OR 1.47. 95% CI 1.13–1.91) [41]. According to the national data from Indonesia’s Central Bureau of Statistics (BPS), there has been an increasing trend of the portion spent on prepared food and beverages, from 29.6% in 2016, to 32.7 and 34% in 2017 and 2018, respectively [42]. There is an established link between the consumption of more food and beverages not prepared at home and higher sodium diet [41]. A lower frequency of eating out may lead to less sodium intake.

In Asian countries, monosodium glutamate (MSG) and sauces are the main products added to the food that contribute to high sodium intake [43]. Healthier options of seasoning should be considered. The consumption of mineral salt rich in magnesium and potassium as a substitute for the regular salt may be beneficial, since it resulted in a significant fall in BP over 8 weeks in a trial with participants having mildly elevated BP [44]. Notwithstanding, caution should be exercised when recommending low-sodium diet to the elderly, due to the possible harmful impact of salt restrictions on this group of people [40].

### Strengths and limitations of the study

To date, we believe that this is the first analysis of multicenter data related to prehypertension in Indonesian middle-aged and older adults involving a large sample size to estimate and represent well the real condition of that population in the nation. We had a population of around 90 million aged 40 years old and above, and 33% of them had prehypertension, which was almost 30 million of middle-aged and older adults had prehypertension. Following the Framingham Heart Study, it is estimated that in the next 4 years, 6 million of the Indonesian prehypertensive population will develop hypertension, which can lead to several complications. This is an alarming condition in Indonesia as it will be present not only medical but also financial burdens to the population and government. Indonesian National Health Insurance stated that the highest number of cases and expenditure was due to hypertension, costing a total of 12.1 billion rupiah during 2014–2016 [45]. Preventive measures as mentioned above need to be taken as soon as possible to tackle the upcoming burden.

Although this is a cross-sectional data analysis, we knew several risk factors such as increased age and male occurred before the incidence of prehypertension. In addition, modifiable risk factors, such as increased BMI and waist circumference have been consistently shown in previous studies related to prehypertension. Data related to pre-existing diseases were only based on history taking which might result in potential recall bias. To overcome the limitations, future studies should follow up the changes of study variables in a longitudinal study to confirm the cause—effect relationship of factors related to prehypertension. Longitudinal studies may involve medical records and additional tests to help confirm the diagnoses reported by the subjects.

## Conclusion

The prevalence of prehypertension in Indonesian mid- and late-life populations was 32.5%. Age ≥ 60 years, male sex, overweight, obesity, and raised waist circumference were risk factors for prehypertension..

## Data Availability

The data used in this study belong to Indonesia Family Life Survey-5 which are accessible via the RAND website: http://www.rand.org/labor/FLS/IFLS.html.

## Abbreviations

IFLS-5: Indonesian family life survey-5
ISH: International Society of Hypertension
OR: Odds ratio
CI: Confidence interval
BP: Blood pressure
SBP: Systolic blood pressure
DBP: Diastolic blood pressure
CVD: Cerebrovascular disease
CAD: Coronary artery disease
CKD: Chronic kidney disease
DM: Diabetes mellitus
IPAQ: International Physical Activity Questionnaire
IPAQ-S7S: International Physical Activity Questionnaire for the last 7 days
BMI: Body Mass Index
WHO: World Health Organization
WRPO: Western Pacific Region of World health organization
HMOD: Hypertension-mediated organ damage
ASCVD: Atherosclerotic cardiovascular disease
SD: Standard deviation
BPS: Indonesia’s Central Bureau of Statistics
MSG: Monosodium Glutamate
